# Technical failure rates for biometry between swept-source and older-generation optical coherence methods: a review and meta-analysis

**DOI:** 10.1186/s12886-023-02926-0

**Published:** 2023-04-26

**Authors:** Piotr Kanclerz, Idan Hecht, Raimo Tuuminen

**Affiliations:** 1 Department of Ophthalmology, Hygeia Clinic, ul. Jaśkowa Dolina 57, Gdańsk, 80-286 Poland; 2 Helsinki Retina Research Group, Faculty of Medicine, University of Helsinki, Helsinki, Finland; 3 Sackler School of Medicine, Tel Aviv University, Tel Aviv, Israel; 4 Department of Ophthalmology, Shamir Medical Center, Tel Aviv, Israel; 5 Eye Centre, Kymenlaakso Central Hospital, Kotka, Finland

**Keywords:** Cataract, Intraocular lens calculation, Low-coherence optical reflectometry, Partial coherence interferometry, Optical biometry, Swept-source optical coherence tomography

## Abstract

**Purpose:**

Precise ocular measurements are fundamental for achieving excellent target refraction following both cataract surgery and refractive lens exchange. Biometry devices with swept-source optical coherence tomography (SS-OCT) employ longer wavelengths (1055–1300 nm) in order to have better penetration through opaque lenses than those with partial coherence interferometry (PCI) or low-coherence optical reflectometry (LCOR) methods. However, to date a pooled analysis showing the technical failure rate (TFR) between the methods has not been published. The aim of this study was to compare the TFR in SS-OCT and in PCI/LCOR biometry.

**Methods:**

PubMed and Scopus were used to search the medical literature as of Feb 1, 2022. The following keywords were used in various combinations: *optical biometry, partial coherence interferometry, low-coherence optical reflectometry, swept-source optical coherence tomography*. Only clinical studies referring to patients undergoing routine cataract surgery, and employing at least two (PCI or LCOR vs. SS-OCT) optical methods for optical biometry in the same cohort of patients were included.

**Results:**

Fourteen studies were included in the final analysis, which presented results of 2,459 eyes of at least 1,853 patients. The overall TFR of all included studies was 5.47% (95% confidence interval [CI]: 3.66–8.08%; overall I^2^ = 91.49%). The TFR was significantly different among the three methods (p < 0.001): 15.72% for PCI (95% CI: 10.73–22.46%; I^2^ = 99.62%), 6.88% for LCOR (95% CI: 3.26–13.92%; I^2^ = 86.44%), and 1.51% for SS-OCT (95% CI: 0.94–2.41%; I^2^ = 24.64%). The pooled TFR for infrared methods (PCI and LCOR) was 11.12% (95% CI: 8.45–14.52%; I^2^ = 78.28%), and was also significantly different to that of SS-OCT: 1.51% (95% CI: 0.94–2.41%; I^2^ = 24.64%; p < 0.001).

**Conclusions:**

A meta-analysis of the TFR of different biometry methods highlighted that SS-OCT biometry resulted in significantly decreased TFR compared to PCI/LCOR devices.

**Supplementary Information:**

The online version contains supplementary material available at 10.1186/s12886-023-02926-0.

## Introduction

Precise measurement of ocular structure distances is essential for excellent refractive outcomes, both in cataract surgery and refractive lens exchange. More than twenty years ago the first optical biometer (IOLMaster®, Carl Zeiss Meditec, Jena, Germany) was introduced; since then several commercially-available instruments have been developed. These devices might employ partial coherence interferometry (PCI), low-coherence optical reflectometry (LCOR), and swept-source optical coherence tomography (SS-OCT) [1–4]. Currently, optical biometry is considered as the gold standard for preoperative biometry.

The first intraocular lens (IOL) calculations formulas were based on three variables that could be extracted from biometry data: axial length, corneal refractive power and the predicted postoperative anterior chamber depth. Newer-generation formulas employ additional parameters to estimate the postoperative effective lens position [5]. Even further modifications are still being made to vergence-based IOL formulas for improved lens power accuracy. The standard deviation of the new-generation IOL calculation formulas in patients undergoing cataract surgery reaches 0.4 D [6], which translates into more than 80% of eyes within 0.5 D target refraction [7]. A single study has even reported ≥ 88% of eyes within 0.5 D target refraction [8]. Not only does this lead to excellence in outcomes, but also increases patient expectations. This is particularly important due to the constantly growing popularity for spectacle-independence with non-toric and toric premium IOLs.

SS-OCT devices employ longer wavelengths (1055–1300 nm), so they are supposed to have better penetration through opaque media than PCI/LCOR devices [9, 10]. However, to date, a pooled analysis showing the technical failure rate (TFR) in these methods has not been published. The aim of this study was to compare the TFR in SS-OCT biometry and PCI/LCOR.

## Methods

PubMed and Scopus were the main resources used to search the medical literature. An extensive look up was performed to identify relevant articles concerning the TFRs in optical biometry as of Feb 1, 2022. The following keywords were used in various combinations: *optical biometry, partial coherence interferometry, low-coherence optical reflectometry, swept-source optical coherence tomography*. Of the studies retrieved by this method, we reviewed all publications in English and abstracts of non-English publications [11]. The search did not aim to find studies designed to report TFR in healthy individuals, [12] but rather to report the rate of technical or capture error in currently published cataract surgery studies. A study was included in this meta-analysis if: (i) it was referring to patients undergoing phacoemulsification cataract surgery, (ii) presented results of a general population, but not only for a subgroup of patients with mature or white cataracts (iii) the study employed at least two or more optical methods for optical biometry in the same cohort of patients: one of them was SS-OCT and the other either PCI, LCOR or both, (iiii) the TFRs for each method used was reported with a 95% confidence interval (CI), or included data to calculate them. Studies were critically reviewed to create an overview and guidance for further search. No attempts to discover unpublished data were made. The search strategy in detail is presented in Supplement 1

TFR was defined as failure in obtaining ocular distance measurements. Such failure could be a result of the presence of dense cataracts, but also posterior subcapsular cataract, macular diseases, staphyloma, vitreous or corneal opacities, and poor fixation [13]. The reason for failure was not relevant as commonly it was not presented in the analyzed study and this study aimed to show real-life results. Devices classified as PCI biometers included the IOLMaster 500 (Carl Zeiss Meditec, Jena, Germany), Galilei G6 (Ziemer Ophthalmic Systems AG, Brügg, Switzerland) and the Pentacam AXL/AXL Wave (OCULUS Optikgeräte GmbH, Wetzlar, Germany), while LCOR biometers were the Lenstar LS900 (Haag-Streit AG, Köniz, Switzerland) and Aladdin (Topcon Corporation, Tokyo, Japan) [14, 15]. SS-OCT biometers which are currently on the market include the IOLMaster 700 (Carl Zeiss Meditec, Jena, Germany), OA-2000 (Tomey Corporation, Nagoya, Japan), Argos (Alcon Laboratories, Inc., Fort Worth, TX, USA), ANTERION (Heidelberg Engineering, Heidelberg, Germany) and Eyestar 900 (Haag-Streit AG, Köniz, Switzerland).

Statistical analysis was performed using Medcalc v. 20.027 (Medcalc Software LTD, Ostend, Belgium). If CIs were not provided within the study, the Wilson method for calculating CIs was applied, as it provides more reliable results than normal approximation with symmetrical confidence intervals [16]. The Freeman–Tukey transformation was employed to calculate the weighted summary proportion. Corresponding heterogeneity (I^2^) estimates were generated, and the I^2^ estimates equal to 25%, 50%, and 75%, were considered as indicating the presence of low, moderate, and high heterogeneity, respectively. As studies did not come from a common population, random effects models were applied. The DerSimonian and Laird approach was employed to calculate the summary proportions under the random effects model. Pooled-analysis was used in order to avoid problems arising from simple pooling. Results with *p* levels under 0.05 were considered statistically significant.

## Results

**Fig. 1:**
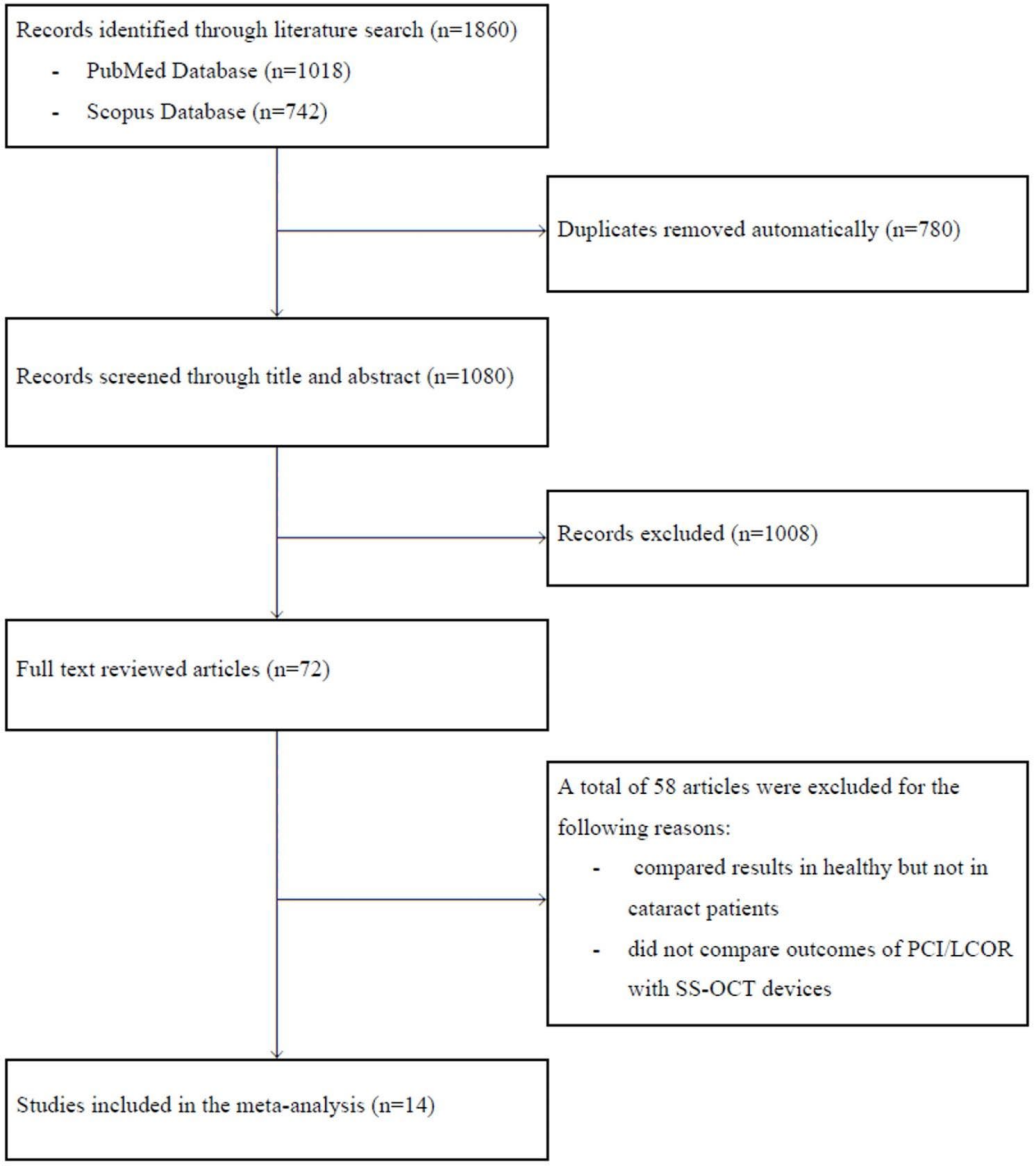
Flow diagram outlining the selection process of the systematic review for on swept-source and older-generation optical coherence methods

The search identified 1,860 eligible publications from the PubMed and Scopus databases; the strategy in detail is presented in Fig. 1 (study chart flow). Several studies have been excluded as they have compared results of optical biometry in healthy but not in cataract patients [17–22], did not compare outcomes of PCI/LCOR with SS-OCT devices [23–29] or both [30]. Finally, fourteen studies were included in the final analysis (Table 1), which presented results of 2,459 eyes of at least 1,853 patients (since in one study [31] only the number of eyes, and not patients was reported).

**Table 1:** Comparative studies showing the technical failure rate for three different optical biometry technologies in patients undergoing routine cataract surgery

Study	Number of eyes / patients	Technical Failure Rate [n] (Device)		
		PCI	LCOR	SS-OCT
Srivannaboon et al. 2015 [24]	100/100	5 (IOLMaster 500)		0 (IOLMaster 700)
Shammas et al. 2016 [32]	107/66	13 (IOLMaster 500)	14 (Lenstar LS900)	2 (Argos)
Kurian et al. 2016 [33]	100/100		21 (Lenstar LS900)	4 (IOLMaster 700)
Akman et al. 2016 [34]	188/101	17 (IOLMaster 500)		0 (IOLMaster 700)
McAlinden et al. 2016 [13]	377/210	136 (IOLMaster 500)	51 (Aladdin)	0 (OA-2000)
Jung et al. 2017 [35]	101/54	7 (Galilei G6)		1 (IOLMaster 700)
Arriola-Villalobos et al. 2017 [36]	80/80		8 (Lenstar LS900)	0 (IOLMaster 700)
Higashiyama et al. 2018 [37]	55/55	7 (IOLMaster 500)		1 (Argos)
Lee and Kim 2018 [38]	175/175	25 (IOLMaster 500)		2 (IOLMaster 700)
An et al. 2019 [39]	431/431	66 (IOLMaster 500)		10 (Argos)
Huang et al. 2019 [40]	171/119	33 (IOLMaster 500)		1 (Argos) 5 (IOLMaster 700) 5 (OA-2000)
Yang et al. 2019 [41]	146/83	17 (IOLMaster 500)		3 (Argos) 3 (IOLMaster 700)
El Chebab et al. 2019 [42]	129/129		1 (Lenstar LS900)	1 (IOLMaster 700)
Cummings et al. 2020 [31]	299/NA		4 (Lenstar LS900)	0 (Argos)

**Abbreviations**: LCOR - low-coherence optical reflectometry, NA - not available, PCI - partial coherence interferometry, SS-OCT - swept-source optical coherence tomography. **Footnote**: no studies (i) fulfilling the inclusion criteria and (ii) evaluating the ANTERION, Pentacam AXL and AXL Wave, or Eyestar 900 could be found

The overall pooled TFR of all included studies was 5.47% (95% CI: 3.66–8.08%; overall I^2^ = 91.49%). The TFR was significantly different among the three methods (p < 0.001): 15.72% for PCI (95% CI: 10.73–22.46%; I^2^ = 99.62%), 6.88% for LCOR (95% CI: 3.26–13.92%; I^2^ = 86.44%), and 1.51% for SS-OCT (95% CI: 0.94–2.41%; I^2^ = 24.64%).

**Fig. 2:**
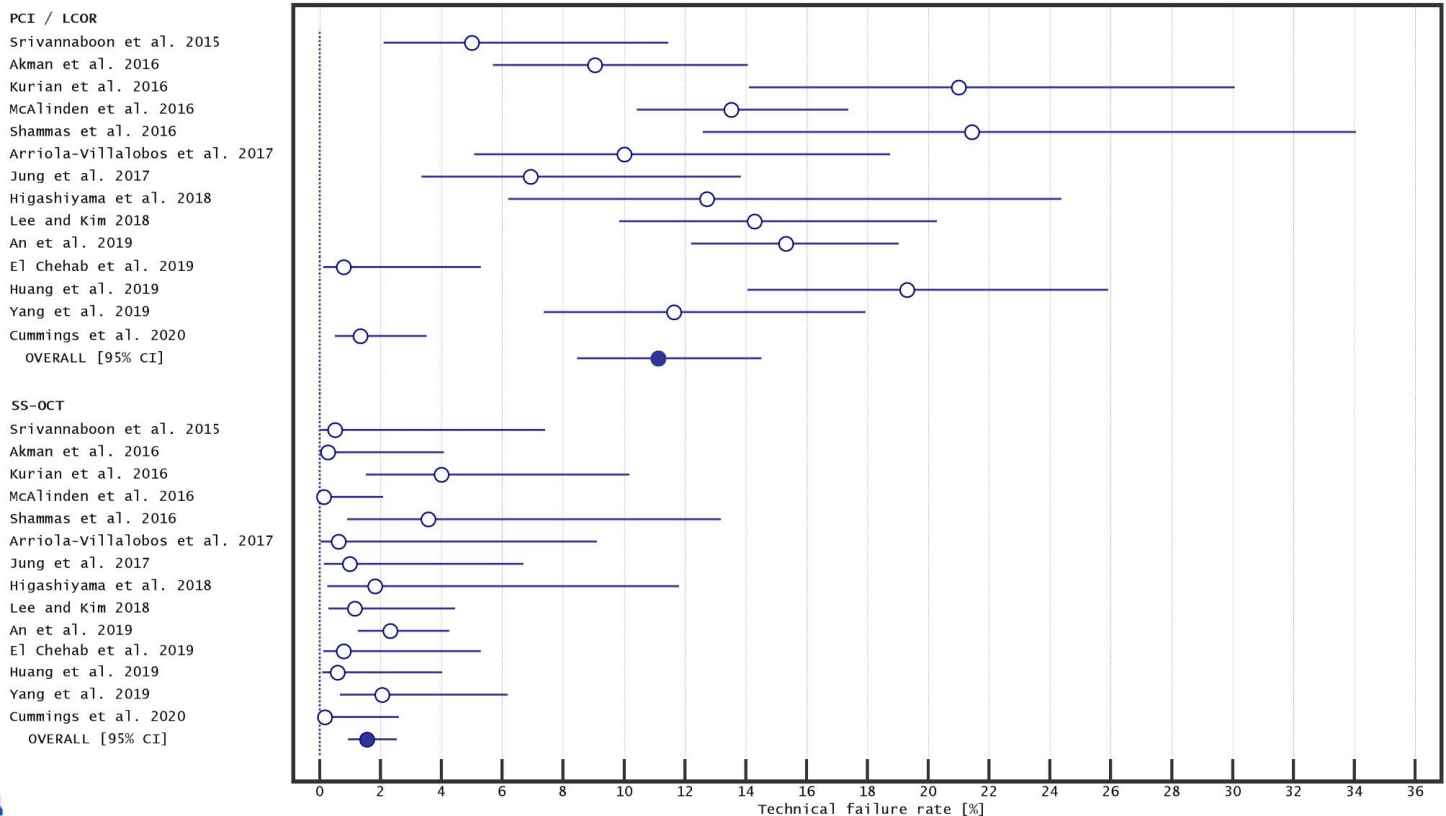
Forest plot showing the technical failure rate in partial coherence interferometry and low-coherence optical reflectometry vs. swept-source optical coherence tomography biometers. Abbreviation: LCOR - low-coherence optical reflectometry, PCI - partial coherence interferometry, SS-OCT - swept-source optical coherence tomography

**Fig. 3:**
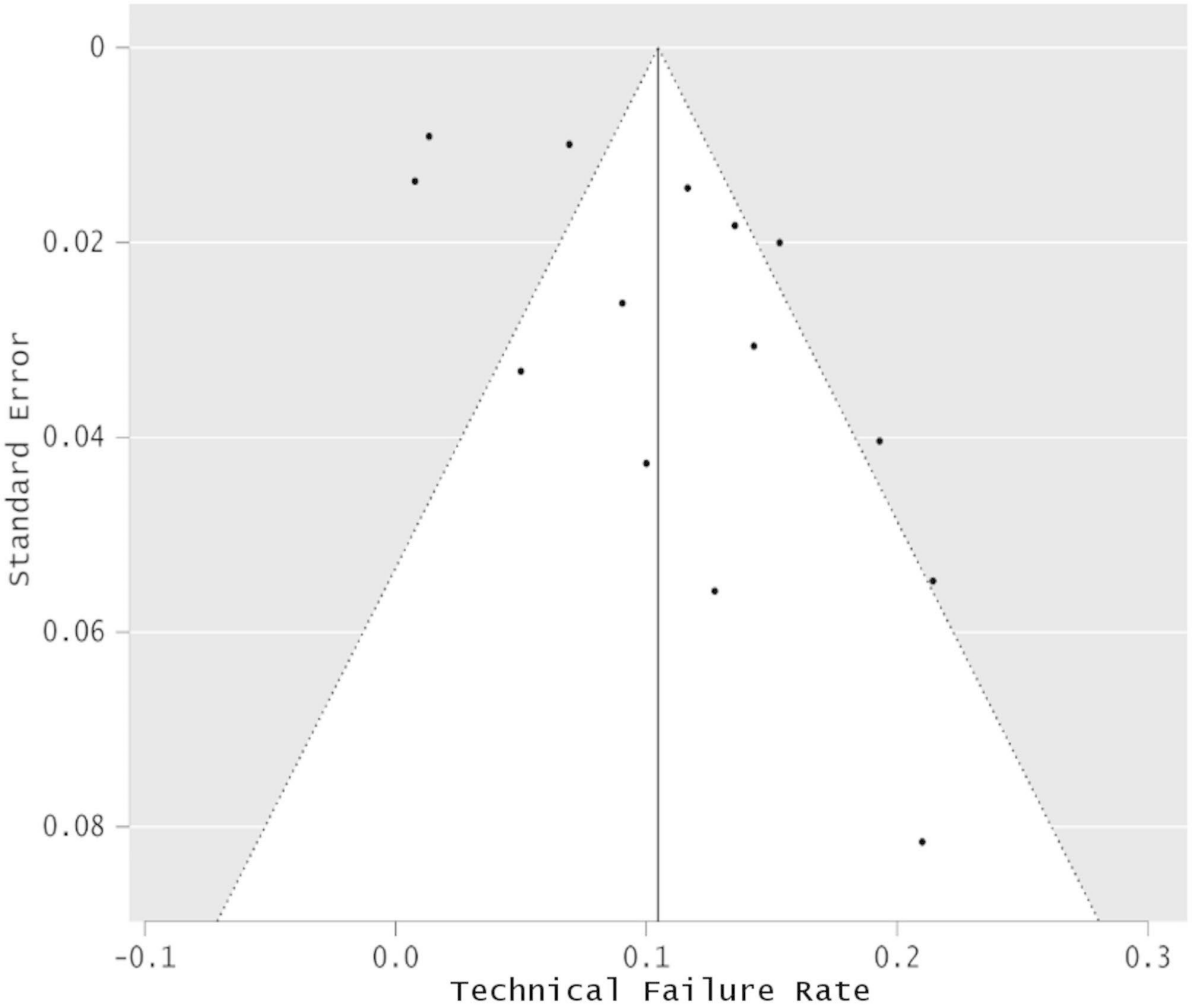
Funnel plot for partial coherence interferometry and low-coherence optical reflectometry biometers

**Fig. 4:**
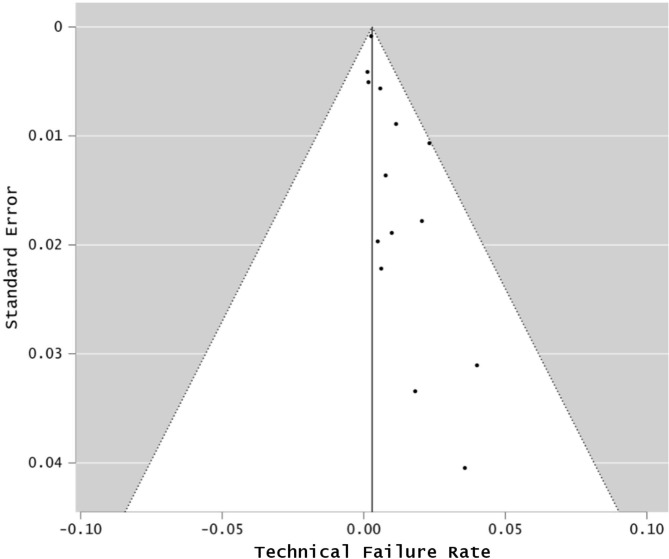
Funnel plot for swept-source optical coherence tomography biometers

The TFR for infrared methods (LCOR and PCI) was 11.12% (8.45–14.52%; I^2^ = 78.28%), and was also significantly different to that of SS-OCT: 1.51% (95% CI: 0.94–2.41%; I^2^ = 24.64%; p < 0.001). A pooled analysis of the included studies is shown in Fig. 2. The funnel plots for PCI/LCOR (Fig. 3) and for SS-OCT (Fig. 4) devices highlighted that several studies regarding PCI/LCOR were situated outside the funnel which confirmed the evidently greater heterogeneity of these studies.

## Discussion

In this review and meta-analysis, the TFR was estimated and compared between different optical biometry methods. Results show that the TFR for infrared methods (LCOR and PCI) used in devices such as the IOLMaster 500, Lenstar LS900 or Aladdin were significantly higher than those using SS-OCT technology, such as the IOLMaster 700, Argos or OA-2000. Not only was the TFR lower among SS-OCT based devices (1.51% vs. 11.12%) but the heterogeneity in reported studies evidently was lower. The highest TFR was seen with devices based on PCI, followed by LCOR based devices, with the lowest rates seen with SS-OCT.

Achieving excellent refractive outcomes following surgery is becoming a common expectation for many patients; in order to achieve this, accurate biometry is essential. Optical biometry is considered superior to other methods, and a high TFR invariably leads to a higher percentage of patients requiring alternative methods to obtain biometry. This could have a negative influence on the refractive outcomes. The differences seen in this study between methods were clinically significant, as an increase from 1.5% to over 10% of patients with insufficient optical biometry can significantly decrease the expected refractive outcomes in any clinic. Considering the efforts routinely used to minimize any residual refraction, the difference seen here could be highly meaningful. The TFR of the biometer is usually unknown and the ocular distances obtained with different biometers cannot be considered interchangeable [18, 19]. Other aspects which are expected to be variable are corneal tomography or the results of IOL calculation made with different formulas; in such a case several measurements or calculations are often conducted and compared before a decision on IOL power is reached. IOL calculations using several biometers based on different technologies is not commonly performed, so the variability in TFR remains an uncontrolled factor that might influence the refractive results.

Phacoemulsification cataract surgery among patients with dense cataracts is associated with increased risk of intra- and postoperative complications e.g., risk of zonulopathy and posterior capsule rupture, greater corneal endothelial damage and prolonged operative time [14, 43–46]. Lower proportion of eyes with dense cataracts achieve excellent uncorrected and best-corrected visual acuity when compared to non-dense cataract [47, 48]. Thus, use of optical biometers with lower TFR might be especially important for patients with dense cataracts which are already prone to other undesirable outcomes. Furthermore, some of the current SS-OCT devices have a retinal visualization mode which can be utilized instead of ultrasound biometry; in this mode, the optical reflex from the retina is enhanced ten-fold [49]. A single study has shown that in cataracts grade IV or higher, according to the Emery-Little classification, the acquisition rates of SS-OCT might range from 63.6% (for IOLMaster 700) to 89.9% (for Argos) [29]. One might estimate that in these advanced cataract cases the TFR might be even higher in LCOR and particularly PCI biometers than in SS-OCT devices. Importantly, the study by Hirnschall et al. has shown that 91.3% of the eyes that were unsuccessfully scanned with PCI IOLMaster 500 were measurable with the SS-OCT IOLMaster 700 [12].

Our study has found a pooled 15.72% TFR (95% CI: 10.73–22.46%; I^2^ = 99.62%) for PCI biometers. This appears very high, keeping in mind that many clinicians are using PCI biometers without any problems on a daily basis; however, this might be dependent on the setting where the device is used and the grading of cataracts. The signal penetration even through a medium-dense cataract in PCI/LCOR devices is lower than with SS-OCT. Not only could this lead to technical failure, but result in a greater variability of the axial length measurements and intraocular lens power calculation predictability. Most of the analyzed studies have reported excellent repeatability and reproducibility in PCI, LCOR, and SS-OCT devices. However, in the study by Kurian et al. the within-subject coefficient of variation in axial length was significantly greater for the LCOR Lenstar LS900 than in the IOLMaster 700 (0.21% vs. 0.05%, respectively) [33]. Jung et al. has shown that the proportion of eyes with an absolute prediction error within 0.5 D was 80.0% for the PCI Galilei G6 and 85.0% for the SS-OCT IOLMaster 700 based on the SRK/T formula [35]. In the study by Yang et al. there was no difference in the percentage of eyes within ± 0.5 D postoperatively between the PCI IOLMaster 500, and SS-OCTs IOLMaster 700 and Argos [41]. The mean absolute prediction error was 0.41 ± 0.31 D, 0.42 ± 0.32 D, and 0.35 ± 0.30 D for IOLMaster 500, IOLMaster 700 and Argos, respectively [41]. Finally, the study by An et al. has shown a trend towards greater postoperative mean absolute error with the PCI IOLMaster 500 than the SS-OCT IOLMaster 700 (0.39 ± 0.30 D vs. 0.36 ± 0.27 D, respectively), but both of the methods outperformed A-scan ultrasound (0.47 ± 0.39 D) [39]. It might be concluded that in cases without a technical failure, the outcome of PCI/LCOR is not inferior to SS-OCT devices [24, 32, 33, 36, 40–42]. A recent review by Montés-Micó et al. reported excellent repeatability and reproducibility in SS-OCT biometers, but was not able to draw conclusions regarding the superiority of SS-OCT when compared to older biometers in terms of precision in IOL calculation [50].

The risk of bias assessment [Supplementary Table 1] represents that several of the studies did not explain in detail the reason for technical failure [13, 24, 31, 34, 36, 38–40]. There are some usability differences between the devices. The time required to acquire measurements might not only affect the patient experience, but could potentially influence the TFR. Although none of the analyzed studies has assessed the difference in acquisition time between the devices, Ruiz-Mesa et al. has shown that IOLMaster 700 measurements, whether they are performed with or without central topography, take less time to perform than older generation optical biometry and corneal tomography with a separate device [51]. It is beyond the scope of this manuscript, but with some devices it might be easier to obtain measurements, particularly if they are automatic rather than semi-automatic in use.

A significant limitation of the meta-analysis is that some studies included results for both eyes [13, 32, 34, 35, 40, 41], while others did not present the number of patients nor the laterality but just the number of eyes [31]. Using a combined measurement from both eyes is likely to be an underestimate of the true variance of a sample [52, 53]. It should also be acknowledged that no comparative studies have been published yet for some new SS-OCT biometers. For example, the ANTERION and the Eyestar 900 biometers have been released just recently (September 2018, and spring 2021, respectively).

In conclusion, in this meta-analysis of the TFR of different optical biometry methods, SS-OCT biometry provided significantly lower TFR compared to devices based on PCI and LCOR. These results could have significant implications on refractive outcomes and highlight the importance of variability in TFR between different devices.

## Electronic supplementary material

Below is the link to the electronic supplementary material.

Supplementary Table 1

PRISMA Checklist

Supplement 1

## Data Availability

The datasets generated and analysed during the current study available from the corresponding author on request.
