# Supplementary material for: Technical failure rates for biometry between swept-source and older-generation optical coherence methods: a review and meta-analysis
Source: BMC Ophthalmol. 2023 Apr 26;23:182. doi: 10.1186/s12886-023-02926-0 (PMC10131302; doi:10.1186/s12886-023-02926-0)
Supplement: Supplementary file 1 — Supplementary Table 1 [file 12886_2023_2926_MOESM1_ESM.doc]

**Supplementary Table 1. Risk of bias assessment in comparative studies showing the technical failure rate for three different optical biometry technologies in patients undergoing routine cataract surgery.**

| **Study** | **Selection bias: allocation concealment** | **Performance bias: blinding of participants and personnel** | **Detection bias: blinding of outcome assessment** | **Attrition bias: incomplete outcome data** | **Reporting bias: selective reporting** |
| --- | --- | --- | --- | --- | --- |
| Srivannaboon et al. 2015 | 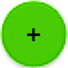 | N/A | N/A | 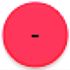 | 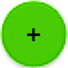 |
| Shammas et al. 2016 | 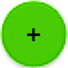 | N/A | N/A | 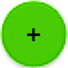 | 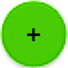 |
| Kurian et al. 2016 | 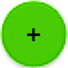 | N/A | N/A | 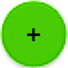 | 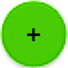 |
| Akman et al. 2016 | 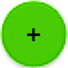 | N/A | N/A | 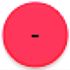 | 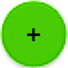 |
| McAlinden et al. 2016 | 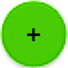 | N/A | N/A | 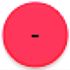 | 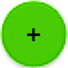 |
| Jung et al. 2017 | 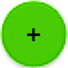 | N/A | N/A | 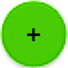 | 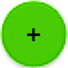 |
| Arriola-Villalobos et al. 2017 | 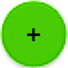 | N/A | N/A | 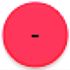 | 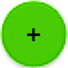 |
| Higashiyama et al. 2018 | 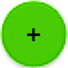 | N/A | N/A | 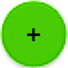 | 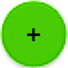 |
| Lee and Kim 2018 | 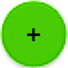 | N/A | N/A | 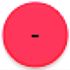 | 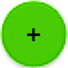 |
| An et al. 2019 | 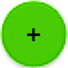 | N/A | N/A | 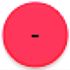 | 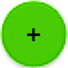 |
| Huang et al. 2019 | 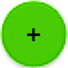 | N/A | N/A | 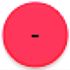 | 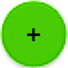 |
| Yang et al. 2019 | 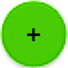 | N/A | N/A | 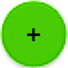 | 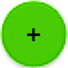 |
| El Chebab et al. 2019 | 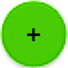 | N/A | N/A | 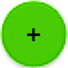 | 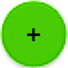 |
| Cummings et al. 2020 | 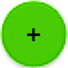 | N/A | N/A | 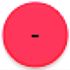 | 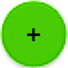 |

**Abbreviations:** LCOR - low-coherence optical reflectometry, N/A - not available/not assessed, PCI - partial coherence interferometry, SS-OCT - swept-source optical coherence tomography
