# Supplementary material for: Technical failure rates for biometry between swept-source and older-generation optical coherence methods: a review and meta-analysis
Source: BMC Ophthalmol. 2023 Apr 26;23:182. doi: 10.1186/s12886-023-02926-0 (PMC10131302; doi:10.1186/s12886-023-02926-0)
Supplement: Supplementary file 3 — Supplement 1 [file 12886_2023_2926_MOESM3_ESM.docx]

**Supplement 1. Search strategy**

Literature searches of the PubMed and Scopus databases were conducted on Feb 1, 2022; the search strategies are as follows. Specific limited update searches were conducted after Feb 1, 2022. Reference lists of the included studies were also considered as a source of publications.

**A.1. PubMed Search (Publication Date 1/10/11–02/01/2022)**

((“*optical biometry*”[Title]) OR (“*partial coherence interferometry*”[Title]) OR (“*low-coherence optical reflectometry*”[Title]) OR (“*swept-source optical coherence tomography*”[Title])). 1018 references.

**A.2. Scopus Search (Publication Date 1/10/11–2/01/2022)**

((“*optical biometry*”[Title]) OR (“*partial coherence interferometry*”[Title]) OR (“*low-coherence optical reflectometry*”[Title]) OR (“*swept-source optical coherence tomography*”[Title])). 742 references.
